# Beauty and Dentistry

**Published:** 1923-10

**Authors:** M. Tew

**Affiliations:** Los Angeles, California


### BEAUTY AND DENTISTRY

BY MISS M. TEW, LOS ANGELES, CALIFORNIA

It is assumed that, as denture prosthetists, you are ready to pledge as loyal allegiance to your artistic sense as to your scientific sense. Being true to both, you can not separate them, nor trust one more than the other. It is a pity we do not have the same feeling of nearness and trust in art that we do in nature. If some of you feel that your artistic sense is too weak to be trusted, you have only to let art help you strengthen it.

Cultivate your sense of beauty in general, and apply it in particular. It is upon the sensitiveness of the nervous system, whether natural or acquired, that the sense of beauty depends, and studying to acquire this sense is a discipline, not an indulgence. Study all growing things, books and great architecture, painting and sculpture, and most of all the faces of your fellow men. Search out *difference* of feature and *similitude*— variety of form and balance of masses. By searching, your discernment will become acute; you will be able to avoid the "almost nothing that will mar everything." Study the harmony of faces; note the homogenity of ugliness as well as the homogenity of beauty. There is a relation between the smallest traits of every part of the face. No one feature of a man could ever be exactly adjusted to the face of another. Learn this great lesson. Nature does not compose a face by matching detached parts—her organizations are not patch-work. Nature operates from the interior to the exterior. The solid parts, independent of the fleshy, are the real bases of drawing and painting. Then comes the study of each facial muscle, its attachments and manner of action, and the resulting effect on the form and expression of the face. Form is the greatest foundation of facial art. You can find hundreds of faces alike in coloring of hair, eyes and skin, but no two alike in form, and it is the form of the mouth and adjacent parts that the greatest individuality lies. No wonder Herder wrote this concerning the mouth: "What a sublime miracle among so many miracles which compose my being! I conjure you, ye painters *and all other artists,* who undertake to represent the figure of man, to study the most precious of all his organs, in all its shades, all its proportions, and all its harmony." Among "all other artists" stands the denture prosthetist, who must do his best to preserve the beauty and usefulness of the mouth, and its harmony with other features. The following passage from an old French book, "De la Plastique," is good for you to hear: "Below the forehead stands that beautiful frontier, the eyebrow, in its mildness, the rainbow of peace; the bended bow of discord when it expresses rage: * * *. The nose combines, and gives a finishing, to all the features of the face; it forms, as it were a mountain of separation between two opposite valleys—the root of the nose, its ridge, its point, the apertures through which it respires life—how many expressive signs of the understanding of character! We have now reached the lower part of the countenance, which nature, in males, surrounds with a cloud, and surely not without reason. * * * It is well known how much the upper lip characterizes the taste, the appetite, the sentiment of love, that pride and anger bend it; that it is sharpened by cunning; that goodness rounds it; that intemperance enervates and debases it; the human figure is nowhere more beautifully and correctly finished, than in the upper lip, at the place where it closes the mouth. The use of the upper lip is to serve as its support. It is, besides, of the greatest importance to observe the arrangement of the teeth, and conformation of the cheeks. A pure and delicate mouth is one of the strongest of recommendations, the beauty of the portal announces the dignity of the passenger; here, that illustrious passenger is the voice. The interpreter of the heart and the soul, the expression of truth, of friendship, and of all tender affections. The upper lip begins already to form the chin, which is terminated by the jawbone, descending on both sides, as it rounds off the whole ellipse of the face, it may be considered as the true keystone which rounds off the arch of the edifice.

By studying some portraits remarkable for harmony, we see how true are the foregoing statements.

Applying your sense to beauty in particular, you will find it most helpful to study *one* mouth *well*—draw and model it fifty times. The mastery of one is the mastery of many. You can model many more, and see those points which are individual and those which are universal. You will develop a dependable sense of line, mass and proportion. Especially study the mouth and adjacent features in profile. The great sculptor Rodin calls the profile the real key to the structure of the head, and the character of man. Some day one of you will surely work out a means of so placing artificial teeth that the profile will not be altered as it now is. In choosing the best color for artificial teeth, a sense of gradation, rather than color, is what you need. Study gradation in nature, in white clouds or gray; in neutral tints such as the bark of trees, shells, stones and stems of plants; the delicate gradation in black and white drawings and etchings.

There are eminent authorities on the medical and physiological aspects of dentition, and some of you should specialize sufficiently to become an authority on the esthetic aspects of both natural and artificial dentition. You probably know that the earliest dentistry practiced in America was on a purely esthetic basis. In pre-Columbian times the natives of Central America operated on the tooth by staining, filing or cutting the ends into different forms, and by the insertion of foreign substances into or through the enamel. Decorative inlays of turquoise, jadeite, obsidian and gold have been found in skulls dug up in Mexico and Yucatan; but among thousands of mummies found in Peru there was not one with filed or inlayed teeth.

De Landa says: "They had the customs of filing the teeth like the teeth of a saw, and this they did for elegance and show; the work was done by women, filing them with certain stones and water." So you see *earliest* authority on esthetic dentistry was a woman, and now it is your turn to be the *latest* authority. If you will study, saying sincerely in your heart "let Beauty awake," Beauty *will* awake, and at the same time will come to you many devices for incorporating beauty in your work.

The following books are recommended for study: Sulzer's "Theory of Fine Arts"; Sir Chas. Bell's "Anatomy of Expression"; Wm. Moris Hunt's "Talks on Art"; Rodin's "The Man and His Art"; Maxfield Parish's and Jules Guerin's book on color; Pater's "Greek Studies"; and Aishoke's "Character Reading Through Features."—*American Dental Journal.*

